# “The Ones that Care Make all the Difference”: Perspectives on Student-Faculty Relationships

**DOI:** 10.1007/s10755-020-09522-w

**Published:** 2020-09-30

**Authors:** Mariana T. Guzzardo, Nidhi Khosla, Annis Lee Adams, Jeffra D. Bussmann, Alina Engelman, Natalie Ingraham, Ryan Gamba, Ali Jones-Bey, Matthew D. Moore, Negin R. Toosi, Sarah Taylor

**Affiliations:** 1 Human Development and Women’s Studies, California State University, East Bay, Hayward, CA 94542 USA; 2 Health Sciences, California State University, East Bay, Hayward, CA 94542 USA; 3 University Libraries, California State University, East Bay, Hayward, CA 94542 USA; 4 Sociology, California State University, East Bay, Hayward, CA 94542 USA; 5 Pioneers for HOPE Learning Framework Program, California State University, East Bay, Hayward, CA 94542 USA; 6 College of Community Health Sciences, University of Nevada, Reno, NV 89557 USA; 7 Psychology, California State University, East Bay, Hayward, CA 94542 USA; 8 Social Work, California State University, East Bay, Hayward, CA 94542 USA

**Keywords:** Student-faculty interactions, Higher education, Accessibility, Retention gap, Underrepresented minorities, Ethics of care

## Abstract

Student-faculty (S-F) interactions that are conducive to students’ learning can help reduce the retention and graduation gaps in higher education, especially for college students from underrepresented and underprivileged backgrounds. The aim of the study was to explore students’ perceptions of their interactions with faculty, and the subjective impact of these interactions on students’ academic and personal life. We analyzed qualitative data from a larger study with the goal of providing best practice models to support students experiencing displacement and food insecurity. Through purposive sampling techniques, 53 students from a diverse public university were recruited. Recruitment strategies focused on students who were likely to be facing academic, personal, and/or financial challenges that may affect their academic performance. Students were interviewed three to four times over a four to six-month period, using semi-structured interview guides. Our multidisciplinary team analyzed data thematically in team-based coding sessions using an online software. We identified four themes for faculty practices: (1) Creating Pedagogical Space, (2) Being Inclusive and Aware, (3) Being Engaged and Engaging Students, (4) Doing More Than Teaching. Based on students’ perspectives, these practices lead to supportive and responsive S-F relationships that facilitate learning and success. The findings have implications related to how faculty can encourage caring S-F relationships and create conducive learning environments where students can thrive, especially during times of crisis.

In a time of rising inequality in the United States (Sommeiller & Price, 2018), a college degree offers individuals one potential path to economic stability (Carnevale, Rose, & Cheah, 2011). Research suggests that while many students are highly motivated to obtain a college degree, many encounter barriers to completion (U.S. Department of Education, Office of Planning, Evaluation, and Policy Development, 2016). The six-year graduation rate at many regional and comprehensive universities hovers around 60% for entering first-year students, with substantial variation between highly selective institutions (87%) and less selective institutions (31%; U.S. Department of Education, National Center for Education Statistics, 2019). These statistics underscore the need to investigate the factors related to this variation in graduation rates.

Several challenges to academic success have been identified in the literature, including food insecurity (Bruening, Argo, Payne-Sturges, & Laska, 2017), homelessness (Goldrick-Rab, Richardson, & Hernandez, 2017), health and mental health issues (Goodman, 2017), the need to work an excessive number of hours while attending school (Blanchard Kyte, 2017), disability (Peña, Stapleton, & Schaffer, 2016), caregiving responsibilities (Wladis, Hachey, & Conway, 2018), and trauma (Jolley, 2017). Given these well-documented challenges, some universities have begun to focus less on whether students are “college-ready” but rather, how universities can be “student-ready” -- that is, how to prepare academic instruction and supports so that all students who matriculate can develop their capacities, regardless of their initial circumstances (McNair, Albertine, Cooper, McDonald, & Major, 2016). To this end, vigorous initiatives are taking place around the country aimed at increasing graduation rates, ranging from changes in registration policies, waitlist policies and advising, to helping students procure basic needs and providing financial assistance (e.g., Wacker, 2017).

Added to the issues affecting retention and degree completion rates, are the complex obstacles related to the COVID-19 pandemic. This crisis, with the resulting economic recession, can exacerbate pre-existing inequities in access, retention and completion of a college degree, particularly for those who are low-income, first-generation or from historically underrepresented groups.

Faculty can play a significant role in counterbalancing structural inequities and contextual challenges by building relationships with students, engaging them in learning, and serving as a conduit to helpful campus resources. Difficult or unsupportive relationships with faculty may serve as an additional hurdle for students who are already feeling overburdened. In our study we focused on students facing academic, personal, and/or financial challenges with the goal of exploring how students perceive their interactions with faculty and the subjective impact of these interactions on engagement in learning. The rationale behind this study is to inform best practices to support students experiencing financial hardship or adverse circumstances during their college career.

In this research, faculty and staff members from multiple disciplines collaborated in analyzing data from a mixed methods longitudinal study of students in a diverse public university. We sought to address the following research questions:

1. How do students perceive their interactions with faculty?
2. From the students’ perspectives, how do these interactions facilitate or hinder their learning and achievement?
3. From the students’ perspectives, how do these interactions impact their well-being as a student?

We examined these questions to better understand our students’ subjective experiences, with the hope that the findings motivate faculty to critically reflect on their own practices so that they can better support student success and achievement.

## The Importance of Student-Faculty Interactions

Extensive research has emphasized the importance of S-F interactions for achieving student success in college (Cuseo, 2018; Kim & Lundberg, 2016; Miller, Williams, & Silberstein, 2019). S-F interactions are positively linked to students’ success, because of their effect on students’ academic achievement, satisfaction with college, intellectual and personal development, persistence and attrition, as well as career and educational aspirations (Cuseo, 2018; Lamport, 1993). In this literature review, we consider S-F interactions in and outside the classroom that influence students’ experiences in higher education. We also discuss the importance of faculty mentoring and caring for students, while being inclusive in their interactions and teaching practices.

### Interactions inside the Classroom Influence Student Achievement

Simple classroom practices that help faculty bond with students in a “personable and empathic manner” (Cuseo, 2018, p. 93) include the following: learning students’ names and using them, timely and constructive assignment feedback (Kim & Sax, 2014), and appropriate faculty disclosures about their own experiences (Cuseo, 2018). These suggestions, and authentic faculty engagement and support of students, are critical to students’ academic success in all disciplines (Ingraham, Davidson, & Yonge, 2018). Success markers for students that can be influenced by positive interactions with faculty include greater academic life satisfaction, lower dropout rates, improved group communication skills, and greater intellectual and personal development (Komarraju, Musulkin, & Bhattacharya, 2010).

### Interactions outside the Classroom Influence Student Achievement

Faculty contact outside the classroom has been linked to higher grade point average (GPA) (Al-Hussami, Saleh, Hayajneh, Abdalkader, & Mahadeen, 2011; Cress, 2008) and has been identified as an independent predictor of student intellectual development, even after controlling for students’ prior educational experiences or abilities (Tinto, 2004). Informal mentoring can also have positive outcomes for students (Hagenauer & Volet, 2014). Previous research finds that early faculty interaction in the first year influences how much mentorship students receive in their senior year, particularly for “average” or “at-risk” students (Fuentes, Ruiz Alvarado, Berdan, & DeAngelo, 2014). However, high-achieving students are more likely to seek mentors, and faculty are more likely to take high achievers under their wings (Fuentes et al.). A successful mentoring S-F relationship is characterized by reciprocity, mutual respect, clear expectations, personal connection, and shared values (Estepp, Velasco, Culbertson, & Conner, 2017). Supporting the development of S-F interpersonal relationships via mentoring is an important strategy for mitigating the effects of a negative campus climate. Cress (2008) found that students who experienced positive relationships with faculty (e.g., feeling respected, receiving advice on academic programs, receiving emotional support, being encouraged to pursue graduate or professional training), were less likely to perceive their campus climate as negative. Feeling marginalized or lacking a sense of belonging can be related to underrepresented students’ departure from the institution (Cole & Griffin, 2013), while positive S-F interaction influenced retention and completion rates. Consequently, the mentoring faculty can contribute to an inclusive learning environment in which students feel welcome, and this will subsequently influence their academic success.

### Diversity and Inclusion within S-F Interactions

Previous research suggests that the nature and extent of S-F interactions are impacted when students and faculty come from different racial/ethnic backgrounds (Cole, 2007; Finley, 2018; Kim & Sax, 2009; Lundberg & Schreiner, 2004; Toosi, Babbitt, Ambady, & Sommers, 2012). Newman’s (2015) findings demonstrate that African American students face discrimination by White faculty, such as getting less academic support than White students or being actively and intentionally discouraged. Students of color report experiencing prejudice and marginalization by faculty, which may consequently relate to a reluctance to approach faculty for mentorship or to doubts about whether they will be able to remain in the institution (Cole & Griffin, 2013). Professors’ prejudices, and the negative S-F interactions that result from them, can be detrimental to students’ self-perception and achievement. According to the *Chronicle of Higher Education*, “Teaching inclusively means embracing student diversity in all forms — race, ethnicity, gender, disability, socioeconomic background, ideology, even personality traits like introversion — *as an asset*” [emphasis added] (2019, para 5). Embracing the diversity of students from an asset-based model is especially important for students of color, given research on their experiences in the college environment.

### Ethics of Care Framework and Study Aim

The ethics of care theoretical framework (Gilligan, 2011; Noddings, 2012) provides insight into how to conceptualize the S-F relationship, one in which, there is a *carer* (professor) and a *cared for* (student). Hawk and Lyons (2008) explain that caring within this framework is an ongoing, relational process that is “concrete” (e.g. actual behaviors and feelings that enhance student learning) and it is influenced by the learner-teacher context. In the ethics of care process (Hawk, 2017; Hawk & Lyons, 2008), *the carer* should be: (1) engrossed with those being cared for, (2) motivated by the needs of the ones being cared for and empathizing with them (not “self-referencing” and considering how one might do or act differently in the same circumstance), (3) committed to the success of the relationship as well as each individual in it, and (4) cognizant that those cared for have the best of intentions and should be viewed in a positive light. Carers in teaching must be “attentive” and “listen” to the experiences of students so that they can understand students’ expressed needs (Noddings, 2012). If faculty intend on truly supporting their students holistically, it must begin with *listening* to the students themselves. The aim of this study was exactly that: to explore students’ perspectives about their interactions with faculty, including what they believe hinders or encourages their learning. We focused on those who were experiencing complex, overlapping personal and academic challenges while attending a university with the majority of students from racial/ethnic underrepresented groups, that serves many non-traditional students.

## Method

We analyzed data from Pioneers for HOPE Learning Framework project, a mixed methods longitudinal needs assessment involving surveys and four waves of open-ended, semi-structured interviews with undergraduate students (*n* = 53 at Wave 1; *n* = 48 at Wave 4) conducted between January and June 2018 at a diverse public university in California. Students’ average age was 26.49 years (SD = 14.54). The Table 1 provides additional sample characteristics.

**Table 1:** Sample Characteristics (*n* = 53)

**Age**	%	#	SD
17–35	76%	40	
Over 35	19%	10	
Not Reported	6%	3	
**Race/Ethnicity**	%	#	
Middle Eastern/Arab	2%	1	
Native American	4%	2	
Asian/Pacific Islander	13%	7	
White/European American	13%	7	
Multiracial	17%	9	
Latinx	19%	10	
Black/African American	23%	12	
Not Reported	9%	5	
**Gender**	%	#	
Male	11%	6	
Female	83%	44	
Non-Binary	2%	1	
Not Reported	4%	2	
**Sexual Orientation**	%	#	
Lesbian	4%	2	
Bisexual	8%	4	
Heterosexual	62%	33	
Other LGBTQ+ Identity	4%	2	
Not Reported	23%	12	
**Expected year of graduation**			
2018	34%	18	
2019	13%	7	
2020	28%	15	
2021	13%	7	
2022 or later	6%	3	
Not Reported	6%	3	
**Average time spent on different activities (hours/week)**			
In doing paid work		15.76	14.04
In doing homework		13.27	9.05
In caregiving		10.80	26.76
In the classroom		9.95	4
**Experiences of food insecurity**			
Worried that food would be insufficient			
Never True	36%	16	
Sometimes True	50%	22	
Always True	5%	2	
Often True	9%	4	
Food purchased was insufficient			
Never True	50%	22	
Sometimes True	36%	16	
Always True	5%	2	
Often True	9%	4	

*Note*. SD = Standard Deviation

The purpose of the needs assessment was to understand barriers and facilitators of student success. The study was funded by a private foundation interested in supporting student success, with a special focus on students experiencing food insecurity, housing instability, or other financial challenges. For a detailed discussion of the overall methods for this project, please see Khosla et al., 2020.

Noting the extensive data in the interviews about students’ perspectives on interacting with faculty, we formed a multi-disciplinary group of faculty members to closely examine this data. Though there was some overlap in the initial project team and the team that analyzed the S-F data for this paper, the S-F paper team was distinct. Through our Office of Faculty Development, we convened a group of 14 faculty and staff from seven academic departments, the library and a campus program aimed at student success (11 of the initial 14 co-authored this paper). The Principal Investigator and Project Coordinator of the initial project team oriented the S-F team to the primary research study and provided access to the already-collected data to the S-F team. The analysis was an iterative process entailing multiple team meetings.

We coded data using Dedoose software for qualitative research (Dedoose Version 8.0.35, 2018). The overall codebook consists of 51 descriptive codes including 30 ‘parent’ codes. Guided by our research questions, we focused on data coded as “student-faculty interactions.” As we began to explore the excerpts through an iterative, team-based process, we identified several sub-categories within “student-faculty interactions” and added 6 ‘child codes’. In addition to coding, Dedoose allows for use of descriptors, which can be used to categorize entire transcripts with participant demographic information and other characteristics. Our team-based approach to the analysis of these data, collected over multiple interviews with students, increased the credibility of our assertions. Faculty from varying disciplinary and personal backgrounds brought their unique perspectives to the analysis and engaged in frequent in-person and email conversations to refine themes and rigorously question preliminary assertions. An ethics of care theoretical lens was considered after the analysis in order to frame the findings (Gilligan, 2011; Noddings, 2012).

## Results

Students provided rich descriptions of interactions that built supportive and responsive relationships with faculty as well as interactions that hindered these. We identified four interrelated themes: (1) Creating Pedagogical Space, (2) Being Inclusive and Aware, (3) Being Engaged and Engaging Students, and (4) Doing More Than Teaching. These themes contributed to building supportive and responsive student-faculty relationships and encompass qualities or behaviors exhibited by professors that make supportive and responsive relationships possible. Students explained how these kinds of relationships with professors in turn led to better learning, achievement, and subjective well-being. Below we provide descriptors for participants alongside their examples and quotes to better illuminate the context better for the readers. We have provided fewer details where confidentiality concerns may arise if detailed descriptors were provided.

### Creating Pedagogical Space

Creating “pedagogical space” means being flexible or willing to adapt course policies and requirements to students’ needs or unique characteristics so that they feel supported in their efforts for academic success. For students with disabilities, it is especially meaningful when faculty are open to, and accepting of, their requests for accommodations. One student (over 35 years old, and from an underrepresented group) explained the emotional significance of a professor’s actions in response to her request for accommodations: “(...) it makes me want to cry because she was so supportive and gave me hope that I could stay in school.” This contrasted with another professor who required documentation of cognitive disability due to a head injury, which this student understood as not believing she was being truthful, and explains that it “upset” her when they required medical records (“they’re not buying that I really was going through that”). Other accommodations mentioned by students as meaningful included adjusting policies on class participation to fit with students’ needs, such as allowing written questions rather than having to ask questions verbally in class for students with anxiety disorders.

Students also discussed how effective, regular communication contributed to a greater level of connection, and to reaching their goals, thus creating pedagogical space. For example, students mentioned the value of additional support to understand class material and clarify instructions. A 30-year old Nursing student explained that she found it helpful when professors would “walk us through the learning, how to take different types of tests and what to expect.” According to students, practices such as these influence their academic achievement.

Adaptability or flexibility is also necessary when students are experiencing distress (e.g., intimate partner problems, issues causing stress or physical health problems, transportation difficulties, etc.), which may prevent students from performing well in class. When professors were less rigid, had flexible policies, and accepted late assignments (or extended deadlines), students explained that it helped them maintain a good GPA and stay on track with their classes. One student (a multiracial female between ages 26–35), for example, explained how extensions gave her “a lot of time to learn and go in depth with (her professor’s) class.” In this way, students connected professors’ flexibility to their retention and success.

#### Reducing the Pedagogical Space

When professors do not demonstrate flexibility, students may feel that they do not fully support them, and this situation can take an emotional toll on students. Thus, pedagogical space can be adversely affected. Students gave examples in which they perceived professors as treating them with rudeness, condescension or disrespect. For example, when discussing a professor’s treatment as patronizing, a 53-year old student in Sociology stated: “I’m an adult, you’re an adult, you can’t talk to me any kind of way.” Some students felt discouraged or lost interest when professors acted arrogantly (e.g., “they are all that”) and seemed “above” answering questions. Similarly, when speaking about email exchanges, students discussed instances in which messages were harshly worded, condescending, accusatory or did not answer their questions, for example, referring the student to the syllabus instead of answering questions directly. These situations may lead students to perceive faculty as an adversary.

Furthermore, the importance of communication for creating pedagogical space extends to the context of online classrooms. While some students explained that online professors can be particularly responsive through email, others reported receiving less support in online classes than they would receive in a face-to-face class. These comments were collected pre-COVID-19, and refer to professors who chose to teach online. With the rapid move to online teaching as a result of the COVID-19 pandemic, these student sentiments are critical for instructors to understand as online teaching continues. Students also discussed significant limitations that may hamper the pedagogical space, such as the lack of an “in-person connection,” having to “self-teach,” and less comfort in asking for letters of recommendation.

According to other students with complex life challenges, a professor’s inflexibility or discouraging tone can impact their ability to do well in class. One example included a 48-year-old student with a documented disability who detailed the difficulty she faced due to a crisis when she needed to assume the role of an emergency childcare provider for a friend’s infant born addicted to methamphetamine, in addition to caring for her own children. After missing deadlines, the student asked the professor for extensions. Even though the professor was initially supportive, at one moment she declined to accept late work and suggested: “You should just drop out of school. You have too much going on.” In the student’s explanation one bears witness to the level of angst this response by a professor caused her during this time:

All my life I could have given up, because the world told me, you ain't worth it. You don’t mean nothing (...) I was like, I don’t understand how you can just go from believing in me to not believing in me.

The student explained that the professor eventually accepted the assignment, but she also stated that the entire incident “almost broke my (...) really, because my spirit is pretty broken. It’s crumbled. I don’t have a lot of support.” This student, who has disabilities, a complicated history of child abuse, and who needed to provide emergency child care for a friend, perceived the professor’s lack of flexibility (i.e., not accepting late work, or requiring documented evidence of health problems) not as an enforcement of policy, but as a case of betrayal.

### Being Inclusive and Aware

Being inclusive and aware relates to student diversity and ensuring that spaces are welcoming for all students. For example, one student (a Sociology major who declined to state their race, ethnicity, or age) presented a positive experience with a professor’s humble approach that valued all students’ comments and opinions:

He's very open minded, very - like, he'll take criticism. But then he also kind of, he - the way he words things really makes us think about it. Like, he doesn't - he never says we're wrong, but he just kind of makes us to, look at it from a different perspective So, gender within itself, seeing how society is evolving right now like is a very big thing. So, that subject within itself brought in a lot of different perspectives from the way one was brought [up], to the culture, to religion, and everything, basically everything that makes us, us.

Students, such as the individual quoted above, discussed interactions with faculty that made them feel like they belong in the class, their perspective is valued, and/or they have the ability to succeed.

#### Consequences of Perceived Exclusion

Some students in our sample unfortunately had interactions with professors that made them feel excluded, or even targeted as incapable. One 30-year old Latinx student felt that a professor linked the shortcomings in the student’s paper to a Latinx background, which unfortunately reaffirmed a stigma of not performing well in school and, as the student explained, is associated with people of the same background. The student clarified that the professor was not targeting Latinx students but had given similar feedback to students from different ethnicities and races.

Then, when I took it the professor, she was just like (...) “You met the requirements, but your writing is bad." (...) and she was like, "Oh, I think it's because you're Latino, you're having Latino people read it. You're Spanish speakers. It's preventing you from writing.” (...) I guess it was a shock to me.

Such statements, relating students’ identity to a lack of capability, are detrimental to their self-concept and can lead to feeling incapable of achieving what other students can accomplish. Unfortunately, there were a few examples, such as this one, of microaggressions or blatant racist acts by professors toward students.

In another example of behavior that makes students feel excluded, a 25- year old Black student recounted a particularly challenging experience in a course. In this class, the professor displayed a video showing violence towards African Americans, which, from the student’s point of view, was provocative and unnecessary. The student explained that the video “sent me over the edge, personally” and would have preferred that the professor taught “without us having to see graphic footage” to demonstrate racism toward African Americans. Additionally, the professor in this same course conducted an in-class activity about ancestry and migration that made the student feel excluded. The person discussed how the design of this activity lacked either sensitivity or competence about the history of slavery in the US, by excluding those whose ancestors were forcibly enslaved and brought to the Americas. When the student approached the professor to address this, the professor dismissed the student’s concerns. Ironically, these were examples of ignorance or racism, the same concepts that the professor may have been trying to discuss in the class.

### Being Engaged and Engaging the Students

Students understandably reported appreciating interactions in which they perceived the professor to be engaged and invested in engaging students. In the words of one 22-year old student in Criminal Justice:

There are some faculty members on this campus that will reach eye to eye to students. They won't patronize them. They wouldn't be like “oh you're just a college student that wants this and that handed to them.” (...) There are professors that actually sit there and listen intently, not just listen and type on their computer while they're doing something else. Those are the professors that I find, they have heart, is what I call it. They have that passion where they want to help students succeed. They're not just there for a paycheck or just there for tenure or whatever it may be. (...) And they don't care about oh you got this many assignments and on time whatever. It's more about do you understand the concept? Do you really think critically about stuff?

This student is describing a level of engagement that is valued and that contributes to a positive experience for the student. In the following paragraphs we elaborate on how students are engaged by professors who challenge (or “push”) them.

#### Being Challenged Contributes to Engagement

Some students appreciated professors who “pushed” (challenged and encouraged) them to work hard, which, according to them, increased the student’s engagement. A 24-year old Philosophy student discussed how professors’ high expectations contribute to his progress and achievement: “they really push you and they really make you work hard (...). Just to really push through the rest of the year and graduate and make sure I’m prepared for the next level.” Many students want to be challenged so that they may reap the benefits of the class material and the professor’s teaching. In contrast, some students shared instances of coursework being so hard that they shifted to another department or lost motivation. Therefore, professors must balance challenging students while keeping the workload manageable.

Lack of engagement by professors can even reduce students’ commitment to their academic goals. Several students discussed how the professor’s lack of passion in the subject material can be disheartening. A student in Business Administration explained that he is making an effort but his “input” is not matched by the professor’s “output” (i.e., feedback, excitement). The student asked “why am I even in school?” showing that the professor’s lack of engagement can be so damaging as to make students feel that their education is meaningless.

### Doing More than Teaching

Students appreciated faculty members when they provided support in a broad way, and did *more* than just teach. Some examples included discussing class concepts during office hours, creating opportunities to explore class concepts in the field, or at other places on campus, advising and mentorship, and demonstrating care for the students’ well-being and academic achievement such as simply listening and being understanding when students approach professors with issues that prevent them from doing well in class. A 21-year old Health Sciences major discussed this desire for “more than teaching”:

I think some faculty or professors need a little bit more help with engaging with the student. Cause a lot of them are just there to teach and then that's it. And that's not really helpful for us and I feel we should be able to go to them to get career advice. They should be able to help give us advice on how to not only be a better student, but in the workforce too, we're gonna need help with that (...) But a lot of professors don't really. They're not really inviting or they don't really provide that for us.

Similarly, other students valued positive interactions that centered around academic advising and career-related advice.

Doing more than teaching has to do with making students feel that they matter. The students know and recognize that professors are dealing with many students, but professors must “make space” and make students feel that the learning of each individual in class is important. A 44-year old student in Human Development explained the need to feel that one matters, and that one can approach professors with questions or concerns:

I realize there are people, they see hundreds and hundreds and hundreds of students but it would be nice if they could deal with each student with a fresh pair [of] ears and a fresh pair of eyes (...) At 44, I'm in a place where I really want to do well. It does not benefit me to lie or to plagiarize or to make it difficult. I'm here to learn (...) You know if the teachers were more approachable, warm, more cordial, then I wouldn't feel so apprehensive about asking them questions.

Students also appreciated professors noticing when they were struggling and referring them to counseling services. Students explained they were more likely to reach out to professors they perceived as warm. Overall, many students mentioned that professors had been instrumental in helping them, especially if support from their family was lacking or not possible. Additionally, approachability and availability, especially conversations outside of the class period, are all qualities in professors that students valued and felt contributed to their academic achievement and enjoyment as learners. A 26-year old student in Psychology has a lengthy discussion of their support needs, which concern faculty who are available full-time, committed to students, and to their job:

I need more tutoring sessions and I need my teachers to be more accessible. We have all these part-time teachers and I never can see them and it sucks because I have so many questions (...) And then I can only meet with them 15 minutes before class or 15 minutes after. And then there's a bazillion other students that are trying to meet with them too. I don't ever have enough access to my teachers and that stresses me out. I need them more involved. I need more full-time teachers.

Given this student’s words, part of *doing more than teaching,* means being available. Effective communication (without condescension or disparagement) and availability are key aspects for student motivation. Without them, student incentive and engagement suffer dramatically.

## Discussion

Our interrelated themes, (1) Creating Pedagogical Space, (2) Being Inclusive and Aware, (3) Being Engaged and Engaging Students, and (4) Doing More Than Teaching, contribute to supportive and responsive S-F relationships, based on students’ perspectives. These findings support previous literature focusing on the importance of the student-faculty relationship to determine student success (Cress, 2008; Kim & Sax, 2009; Miller et al., 2019), and emphasize particular aspects of these interactions that are decidedly relevant for students facing particular financial hardship (Hagenauer & Volet, 2014). Our findings also contribute to the very limited literature on the importance of flexible pedagogy that applies to first generation, low-income students in the United States.

It is worth noting that our findings reflect the experiences of students who are committed to making it through college, despite not having enough food or secure housing, and often having to deal with racism, sexism, and ableism. The outcomes of this group of students may depend much more on the quality of their relationships with faculty than a comparison set of students who do not face these challenging circumstances. For these students, we suggest, the stakes of college are exceedingly high, and this makes it vital to ensure that members of the faculty and administration are aware of their needs and their perceptions of what facilitates or hinders their success.

### Implications

An ethics of care theoretical lens applied to teaching in higher education (Hawk, 2017; Hawk & Lyons, 2008; Keeling, 2014) resonates with our findings and helps underscore several implications for our work:

1. When we consider how to create pedagogical space by allowing flexibility taking into consideration students’ characteristics and individual needs, we must start with the ethics of care approach of *listening*. After the professor carefully listens and considers the student’s needs, they must consider how to respond positively, and if this is not possible (e.g., lacking the resources to do so), the objective becomes maintaining that caring relation (e.g., keeping lines of communication open). When reacting to a need for accommodations, we also follow the ethics of care principle: the assumption that the student has the best of intentions. Faculty can be flexible while maintaining academic rigor based on what makes pedagogical sense. Casey and Wilson (2005) created a flexibility grid that can be a helpful tool in assessing the current flexibility of a course and potential areas for increased flexibility in course design. This calls for a sense of coherence between flexibility and rigor, rather than seeing these two principles as in conflict. Consequently, faculty can use their pedagogical tools effectively to help students identify and refine their own inherent capacity and skills.
2. Using an ethics of care approach, the professor should be committed to the success of the relationship as well as to each individual, understand the student as a unique individual, and respect each one. Additionally, it is necessary to recognize that each person in this S-F relationship has a role to play: the students have needs and expectations and it is their right to get them fulfilled, and the teacher must evaluate the student as well as evaluate their own performance. Professors can make students feel included by demonstrating a sensitivity to their backgrounds, and forethought about how they might be affected by the teaching material. Doing an anonymous survey about students’ backgrounds at the beginning of a class can be a simple way to address this, supplemented with teaching materials and in-class activities that reflect an awareness of the experiences of diverse groups’ experiences. Furthermore, the way in which we conceptualize the experiences which shape students’ lives has a considerable impact, as noted by Zerquera, Ziskin, and Torres (2018). Rather than perceiving nontraditional or racially diverse students as facing *barriers*, faculty should perceive them as resilient, and take a more assets-based approach (i.e. considering students’ characteristics as strengths rather than inadequacies). Additionally, we may be unintentionally reproducing the same pedagogies that are most often attuned to the needs of traditional students, and that may carry over biases. Following the ethics of care perspective, we should not only be motivated by the students’ needs, but also continually work on empathizing with them. One way of accomplishing this is continually participating in professional development related to inclusive practices and workshops on implicit bias and anti-racism strategies in higher education.
3. Ethics of care within the S-F relationship also entails having respect toward “the integrity of both parties within the caring relationship and for the constructive developmental process or journey of both parties to the caring relationship” (Hawk & Lyons, pp. 322–323). In an effort to contribute to the integrity of the S-F relationship and to consider students’ perspectives, it is necessary to receive feedback from mid-term evaluations, formative evaluations, and peer feedback. These can give faculty useful tips, not only on assessing how engaged students perceived their professors to be, but also reveal students’ impressions and feelings regarding the particular classroom environment. There may be additional benefits such as students believing that their opinions are valued and recognizing that the faculty is engaging in self-reflection and improvement.
4. In an ethics of care, *the carer* or professor needs to go past teaching the subject matter of the class, and focus on an “expressed need of the student for emotional support, moral direction, or shared human interest” (Noddings, 2012). After listening to students’ expressed challenges (engrossed with the person being cared for), responding within an ethics of care entails thinking through the issues or experiences that students bring up and having a dialogue with them (Noddings). When students experience struggles, we must accept our responsibility to act on what we notice and prepare (even preemptively) to respond; this acknowledges human connectedness and interdependence (Keeling, 2014). Demonstrating that one is ‘doing more than teaching’ perhaps reflects specifically the needs of students that may not have supportive family backgrounds or for other reasons, need special mentoring and support. Showing concern for students, encouraging them and making the intent behind pedagogical decisions clear can help build this aspect of S-F interactions. The level of the faculty’s approachability is key: “the approachability of lecturers is relevant not only for TSR [the teacher-student relationship], but also for an overall feeling of connectedness to the university and preventing students from becoming alienated from the university” (Hagenauer & Volet, 2014, p. 378).
5. Though our data was collected before the pandemic, it is clear that teaching in the wake and aftermath of COVID-19 must entail trauma-informed practices. This approach requires being aware of the potential underlying causes of behaviors and whether students need support. It also requires being knowledgeable about how to respond to trauma with students, and creating a caring, safe and flexible classroom environment in which faculty direct students to resources on campus as needed (Gutierrez & Gutierrez, 2019).

These are actions that faculty can take to model and cultivate an ethics of care within our profession and possibly build more supportive and responsive S-F relationships. Our team took direct action as a result of these findings, creating online instructional modules on topics ranging from accessibility in the classroom to serving a diverse student body, as well as a handout about supporting students’ resilience that was distributed to 800 faculty on our campus.

Our study’s strengths include first, our longitudinal data collection, with questions based on emergent issues. Second, we received much-needed perspectives from many participants that represented what were considered ‘non-traditional students’, highlighting their unique voices and lenses. A limitation of this study is that our findings do not include faculty’s perspectives involved specifically in the S-F interactions discussed. While we honor students’ voices, it is possible that in some instances, students may have misunderstood pedagogical considerations, faculty tone or intent, or course policies. We should note that there are contextual factors possibly influencing students’ perceptions. For example, the ‘transactional’ nature of today’s educational environment may have implicitly or explicitly led some students to automatically ‘expect’ flexibility as a right that comes from paying for their education, rather than understanding the necessity to demonstrate their ‘need’ for flexibility or alternative arrangements to their professor(s). Additionally, when considering students’ perceptions regarding limited office hours of some faculty, we acknowledge that students may be unaware that contingent faculty (lecturers/ad hoc faculty) hired to teach specific courses only, are not expected to be available extensively to advise students.

## Conclusion

The S-F interactions that students have described within this study suggest a need for faculty professional development that encompasses a broad spectrum of concepts related to ethics of care theory, an assets-based approach that values students’ inherent capacity, inclusive practices that consider equity issues and adverse life circumstances, and refining pedagogical tools to more effectively enable students to identify and cultivate their skills. An ethics of care approach that includes trauma-informed practices in teaching is particularly important during and post-crisis, such as the COVID-19 pandemic. These conclusions are supported by our study based on students’ perspectives. Students’ perceptions of their experiences with faculty are invaluable to understanding how we can best teach and support them, and take proactive steps towards pedagogical and institutional changes, with the ultimate goal of students achieving academic success.

## Data Availability

The datasets used and analyzed during the current study are available from the corresponding author on reasonable request.
